# Large-Scale Release of *Campylobacter* Draft Genomes: Resources for Food Safety and Public Health from the 100K Pathogen Genome Project

**DOI:** 10.1128/genomeA.00925-16

**Published:** 2017-01-05

**Authors:** Allison M. Weis, Bihua C. Huang, Dylan B. Storey, Nguyet Kong, Poyin Chen, Narine Arabyan, Brent Gilpin, Carl Mason, Andrea K. Townsend, Woutrina A. Smith, Barbara A. Byrne, Conor C. Taff, Bart C. Weimer

**Affiliations:** a School of Veterinary Medicine, UC Davis, Davis, California, USA; b Institute of Environmental Science & Research Ltd., Christchurch, New Zealand; c Department of Enteric Diseases, Armed Forces Research Institute of Medical Sciences (AFRIMS), Bangkok, Thailand; d Department of Wildlife, Fisheries and Conservation Biology, UC Davis, Davis, California, USA; e 100K Pathogen Genome Project, UC Davis, Davis, California, USA

## Abstract

*Campylobacter* is a food-associated bacterium and a leading cause of foodborne illness worldwide, being associated with poultry in the food supply. This is the initial public release of 202 *Campylobacter* genome sequences as part of the 100K Pathogen Genome Project. These isolates represent global genomic diversity in the *Campylobacter* genus.

## GENOME ANNOUNCEMENT

*Campylobacter* is the most common foodborne pathogen worldwide in humans and animals (1). Approximately 1.3 million people are infected in the United States yearly (2). Despite control efforts, outbreaks are increasing (2). Symptoms include fever, abdominal cramping, and bloody diarrhea, and in rare cases, infection leads to autoimmune disorders, such as Guillain-Barré syndrome (8). Infection is primarily from poultry but is also associated with domesticated livestock (3, 4) and wildlife (5–7).

The 100K Pathogen Genome Project (http://www.100kgenomes.org) is a large-scale sequencing effort for worldwide isolates with a genome repository at the 100K Project BioProject at the NCBI (PRJNA186441). This project included the three most commonly identified species of *Campylobacter*: *C. jejuni*, *C. lari*, and *C. coli*. For an in-depth review of *Campylobacter* molecular biology and pathogenesis, see the papers by Silva et al. (9) and Young et al. (10).

All *Campylobacter* isolates from the 100K Pathogen Genome Project were collected and banked in the laboratory of Bart Weimer (University of California, Davis, Davis, CA). Isolates were checked for purity and stored in liquid nitrogen (11). Genomic DNA (gDNA) was extracted from cultures grown on 5% blood agar plates (UC Davis, VetMed Biological Services) for one to two days, lysed (12), purified with a Qiagen QIAamp DNA minikit (catalog no. 51306), and analyzed on Agilent 2200 TapeStation system with the Genomic DNA ScreenTape assay for integrity of gDNA (13). After isolation, gDNA was fragmented using Diagenode Bioruptor next-generation sequencer (NGS) or Covaris E220 (14). Fragmented gDNA (1 μg) was used for library construction with the Kapa high-throughput (HTP) library preparation kit (catalog no. KK8234; Kapa Biosystems, Boston, MA), using the Agilent Bravo NGS workstation (Santa Clara, CA). Fragmented double-strand gDNA molecules were end-repaired (5′), adenylated (3′), and ligated with double-stranded DNA (dsDNA) adapters, either NEXTflex-96 DNA barcode (Bioo Scientific, Austin, TX), and multiplexed up to 96 isolates, or using Integrated DNA Technologies, Inc. Weimer 384 TS-LT DNA barcodes that allowed multiplexing up to 384 genomes. The standard Kapa protocol with dual-SPRI size selection was used for 250- to 500-bp fragments. Library amplification was done for eight cycles using the Kapa HiFi HotStart ReadyMix, followed by a 1× SPRI bead cleanup step. Size distribution of libraries was confirmed using Agilent 2100 Bioanalyzer system with high-sensitivity DNA kit (15, 16), and indexed libraries were quantified with quantitative PCR (qPCR)-based Kapa library quantification kit (catalog no. KK4824) prior to pooling for sequencing either on the Illumina HiSeq 2000 with PE100 plus index read at BGI@UCD (Sacramento, CA) or PE150 on the Illumina HiSeq 3000 at the UC Davis Genome Center (Davis, CA). Paired-end reads were assembled using ABySS 1.5.2 using *k* = 64 (17).

All sample sequences are publicly available on the NCBI’s Sequence Read Archive (SRA) database (http://www.ncbi.nlm.nih.gov/sra), and genome assemblies can be found in NCBI GenBank. Here, the 100K Pathogen Genome Project has assembled 202 genomes from different isolates of *Campylobacter* identified as *C. jejuni* (167 genomes), *C. coli* (32 genomes), and* C. lari* (3 genomes) (Table 1).

**TABLE 1:** Species, isolate name, assembly data, and accession numbers of 202 *Campylobacter* genomes

GenBank accession no.	Isolate name	Species^a^	No. of contigs	Total genome size (bp)	No. of CDSs^b^
MJWB00000000	BCW_4453	*C. coli*	34	1,746,170	1,766
MJVZ00000000	BCW_4454	*C. coli*	46	1,983,591	2,062
MJWE00000000	BCW_4455	*C. coli*	43	1,872,803	1,937
MJWC00000000	BCW_4457	*C. coli*	37	1,834,436	1,893
MKAY00000000	BCW_4724	*C. coli*	31	1,711,767	1,724
MKCC00000000	BCW_5137	*C. coli*	25	1,725,970	1,742
MKAO00000000	BCW_5818	*C. coli*	48	1,837,036	1,907
MJYL00000000	BCW_5914	*C. coli*	53	1,819,402	1,890
MJWD00000000	BCW_5916	*C. coli*	32	1,828,621	1,894
MJWF00000000	BCW_5917	*C. coli*	37	1,823,469	1,893
MJVY00000000	BCW_5918	*C. coli*	40	1,964,242	2,043
MJWL00000000	BCW_6447	*C. coli*	32	1,861,852	1,912
MJWM00000000	BCW_6448	*C. coli*	31	1,857,061	1,912
MJWA00000000	BCW_6450	*C. coli*	48	1,983,760	2,061
MJYP00000000	BCW_6860	*C. coli*	48	1,731,589	1,766
MJZG00000000	BCW_6913	*C. coli*	41	1,812,371	1,882
MJZH00000000	BCW_6914	*C. coli*	43	1,827,738	1,884
MKEX00000000	BCW_6946	*C. coli*	46	1,828,827	1,893
MJZK00000000	BCW_6948	*C. coli*	35	1,740,538	1,767
MJZJ00000000	BCW_6949	*C. coli*	43	1,823,646	1,888
MJZL00000000	BCW_6950	*C. coli*	34	1,740,481	1,766
MJZM00000000	BCW_6951	*C. coli*	57	1,966,450	2,056
MJZP00000000	BCW_6955	*C. coli*	41	1,818,341	1,886
MJZR00000000	BCW_6957	*C. coli*	47	1,868,605	1,928
MJZS00000000	BCW_6958	*C. coli*	40	1,738,043	1,774
MJZU00000000	BCW_7432	*C. coli*	35	1,874,299	1,934
MJZV00000000	BCW_7433	*C. coli*	41	1,813,482	1,882
MJZW00000000	BCW_7434	*C. coli*	48	1,848,847	1,926
MJZX00000000	BCW_7435	*C. coli*	62	1,967,893	2,051
MJZY00000000	BCW_7437	*C. coli*	41	1,829,683	1,892
MKAA00000000	BCW_7460	*C. coli*	36	1,629,723	1,686
MKAB00000000	BCW_7692	*C. coli*	51	1,785,336	1,805
MEIB00000000	BCW_3781	*C. jejuni*	59	1,803,513	1,853
MJYK00000000	BCW_3782	*C. jejuni*	41	1,669,259	1,677
MEIC00000000	BCW_3784	*C. jejuni*	30	1,748,672	1,864
MJWO00000000	BCW_3785	*C. jejuni*	28	1,731,393	1,759
MJWP00000000	BCW_3786	*C. jejuni*	76	1,808,881	1,856
MJVF00000000	BCW_3787	*C. jejuni*	75	1,805,330	1,856
MJVG00000000	BCW_3788	*C. jejuni*	28	1,673,630	1,695
MJVH00000000	BCW_3789	*C. jejuni*	31	1,681,150	1,765
MJVI00000000	BCW_3790	*C. jejuni*	75	1,809,651	1,836
MJVJ00000000	BCW_3791	*C. jejuni*	141	2,006,566	2,034
MJVK00000000	BCW_3792	*C. jejuni*	74	1,809,576	1,833
MJVL00000000	BCW_3794	*C. jejuni*	63	1,759,193	1,775
MJVM00000000	BCW_3797	*C. jejuni*	31	1,699,040	1,676
MJVN00000000	BCW_3798	*C. jejuni*	53	1,744,518	1,751
MJVO00000000	BCW_3799	*C. jejuni*	55	1,768,431	1,783
MJVP00000000	BCW_3800	*C. jejuni*	70	1,832,983	1,840
MJVQ00000000	BCW_3802	*C. jejuni*	64	1,836,234	1,848
MJVR00000000	BCW_3803	*C. jejuni*	19	1,693,605	1,689
MKAL00000000	BCW_3804	*C. jejuni*	42	1,700,860	1,688
MJVS00000000	BCW_3805	*C. jejuni*	74	1,807,158	1,832
MJVT00000000	BCW_3807	*C. jejuni*	95	1,997,747	2,027
MJVU00000000	BCW_3810	*C. jejuni*	67	1,827,740	1,836
MJVV00000000	BCW_4216	*C. jejuni*	29	1,689,963	1,694
MKAM00000000	BCW_4218	*C. jejuni*	36	1,688,993	1,707
MJXR00000000	BCW_4219	*C. jejuni*	29	1,689,256	1,693
MJXS00000000	BCW_4220	*C. jejuni*	71	1,812,101	1,834
MJWI00000000	BCW_4221	*C. jejuni*	33	1,660,182	1,633
MJXT00000000	BCW_4222	*C. jejuni*	76	1,812,008	1,832
MJXU00000000	BCW_4223	*C. jejuni*	73	1,792,952	1,790
MJWH00000000	BCW_4224	*C. jejuni*	29	1,680,989	1,689
MJXV00000000	BCW_4225	*C. jejuni*	72	1,808,664	1,827
MJXW00000000	BCW_4226	*C. jejuni*	29	1,693,524	1,706
MJXX00000000	BCW_4228	*C. jejuni*	74	1,776,092	1,777
MJXY00000000	BCW_4229	*C. jejuni*	76	1,777,005	1,776
MJWJ00000000	BCW_4230	*C. jejuni*	34	1,670,410	1,639
MJXZ00000000	BCW_4231	*C. jejuni*	66	1,833,811	1,842
MKAW00000000	BCW_4317	*C. jejuni*	87	1,729,089	1,758
MKAQ00000000	BCW_4319	*C. jejuni*	18	1,662,736	1,639
MJYA00000000	BCW_4321	*C. jejuni*	67	1,840,105	1,882
MJYB00000000	BCW_4322	*C. jejuni*	67	1,806,657	1,837
MJYC00000000	BCW_4323	*C. jejuni*	88	1,736,882	1,756
MKET00000000	BCW_4324	*C. jejuni*	56	1,804,461	1,856
MJYD00000000	BCW_4325	*C. jejuni*	88	1,733,375	1,755
MJYE00000000	BCW_4326	*C. jejuni*	90	1,732,137	1,750
MKAN00000000	BCW_4328	*C. jejuni*	63	1,854,445	1,889
MJYF00000000	BCW_4332	*C. jejuni*	70	1,739,132	1,764
MJYG00000000	BCW_4333	*C. jejuni*	81	1,874,835	1,927
MJYH00000000	BCW_4335	*C. jejuni*	91	1,725,603	1,756
MJYI00000000	BCW_4337	*C. jejuni*	68	1,806,323	1,836
MJYJ00000000	BCW_4338	*C. jejuni*	66	1,807,200	1,840
MKAH00000000	BCW_4341	*C. jejuni*	55	1,773,112	1,773
MJWG00000000	BCW_4452	*C. jejuni*	25	1,800,178	1,850
MKAI00000000	BCW_4456	*C. jejuni*	30	1,724,619	1,726
MJWN00000000	BCW_4459	*C. jejuni*	55	1,942,729	2,031
MKAJ00000000	BCW_4460	*C. jejuni*	25	1,601,734	1,576
MKAK00000000	BCW_4461	*C. jejuni*	22	1,604,851	1,577
MKAZ00000000	BCW_4727	*C. jejuni*	13	1,678,985	1,684
MKBA00000000	BCW_4728	*C. jejuni*	41	1,712,512	1,743
MKBB00000000	BCW_4731	*C. jejuni*	32	1,698,127	1,731
MKBC00000000	BCW_4734	*C. jejuni*	34	1,684,111	1,732
MKBD00000000	BCW_4735	*C. jejuni*	35	1,837,130	1,892
MKBE00000000	BCW_4737	*C. jejuni*	36	1,642,302	1,692
MKBF00000000	BCW_4738	*C. jejuni*	40	1,748,256	1,788
MKBG00000000	BCW_4741	*C. jejuni*	172	1,819,989	1,970
MKBH00000000	BCW_4743	*C. jejuni*	18	1,644,315	1,648
MKBI00000000	BCW_4744	*C. jejuni*	61	1,654,980	1,715
MKBL00000000	BCW_4747	*C. jejuni*	22	1,718,223	1,720
MKBJ00000000	BCW_4748	*C. jejuni*	49	1,747,262	1,782
MKBK00000000	BCW_4749	*C. jejuni*	48	1,704,441	1,757
MKBM00000000	BCW_4753	*C. jejuni*	60	1,661,715	1,720
MKBN00000000	BCW_4755	*C. jejuni*	27	1,687,997	1,681
MKBO00000000	BCW_4757	*C. jejuni*	51	1,645,272	1,708
MKBP00000000	BCW_5121	*C. jejuni*	52	1,891,211	1,916
MKBQ00000000	BCW_5122	*C. jejuni*	94	1,846,320	1,949
MKBR00000000	BCW_5123	*C. jejuni*	60	1,722,506	1,797
MKBS00000000	BCW_5124	*C. jejuni*	66	1,673,066	1,727
MKBT00000000	BCW_5125	*C. jejuni*	49	1,774,518	1,808
MKBU00000000	BCW_5126	*C. jejuni*	69	1,815,843	1,883
MKBV00000000	BCW_5128	*C. jejuni*	116	1,797,324	1,868
MKBW00000000	BCW_5129	*C. jejuni*	58	1,703,448	1,777
MKBX00000000	BCW_5131	*C. jejuni*	120	1,775,968	1,880
MKBY00000000	BCW_5132	*C. jejuni*	38	1,694,325	1,735
MKBZ00000000	BCW_5133	*C. jejuni*	140	1,858,971	1,987
MKCA00000000	BCW_5135	*C. jejuni*	105	1,776,102	1,902
MKCB00000000	BCW_5136	*C. jejuni*	52	1,780,266	1,796
MKCD00000000	BCW_5140	*C. jejuni*	42	1,691,709	1,726
MKCE00000000	BCW_5141	*C. jejuni*	73	1,701,529	1,760
MKCF00000000	BCW_5143	*C. jejuni*	95	1,795,032	1,848
MKCG00000000	BCW_5144	*C. jejuni*	95	1,787,787	1,880
MKCH00000000	BCW_5145	*C. jejuni*	65	1,752,372	1,798
MKCI00000000	BCW_5146	*C. jejuni*	60	1,764,810	1,832
MKCJ00000000	BCW_5147	*C. jejuni*	130	1,721,583	1,824
MKCK00000000	BCW_5148	*C. jejuni*	42	1,670,278	1,703
MKCL00000000	BCW_5150	*C. jejuni*	98	1,794,347	1,876
MKCM00000000	BCW_5151	*C. jejuni*	53	1,683,276	1,727
MKEY00000000	BCW_5152	*C. jejuni*	41	1,685,669	1,721
MKEZ00000000	BCW_5154	*C. jejuni*	65	1,741,318	1,809
MKFA00000000	BCW_5155	*C. jejuni*	67	1,722,407	1,807
MKFB00000000	BCW_5156	*C. jejuni*	85	1,817,799	1,814
MKFC00000000	BCW_5157	*C. jejuni*	88	1,810,646	1,803
MKFD00000000	BCW_5158	*C. jejuni*	26	1,643,431	1,670
MKFE00000000	BCW_5159	*C. jejuni*	40	1,633,952	1,655
MKFF00000000	BCW_5160	*C. jejuni*	54	1,824,739	1,887
MKHS00000000	BCW_5161	*C. jejuni*	56	1,770,897	1,786
MKHT00000000	BCW_5162	*C. jejuni*	34	1,771,963	1,791
MKHU00000000	BCW_5166	*C. jejuni*	47	1,880,441	1,915
MKHV00000000	BCW_5167	*C. jejuni*	32	1,715,657	1,746
MKHW00000000	BCW_5170	*C. jejuni*	68	1,803,925	1,863
MKHX00000000	BCW_5171	*C. jejuni*	117	1,764,157	1,809
MKHY00000000	BCW_5172	*C. jejuni*	64	1,695,537	1,706
MKHZ00000000	BCW_5174	*C. jejuni*	98	1,757,175	1,779
MKES00000000	BCW_5913	*C. jejuni*	80	1,829,124	1,791
MJXA00000000	BCW_6451	*C. jejuni*	23	1,658,447	1,671
MJXB00000000	BCW_6452	*C. jejuni*	25	1,657,388	1,670
MJXC00000000	BCW_6453	*C. jejuni*	73	1,870,366	1,907
MJXD00000000	BCW_6454	*C. jejuni*	78	1,839,000	1,885
MJXE00000000	BCW_6456	*C. jejuni*	47	1,738,922	1,802
MJXF00000000	BCW_6457	*C. jejuni*	66	1,820,178	1,841
MJXG00000000	BCW_6458	*C. jejuni*	48	1,770,748	1,779
MJXH00000000	BCW_6459	*C. jejuni*	91	1,873,732	1,895
MJXI00000000	BCW_6460	*C. jejuni*	42	1,743,085	1,756
MJXJ00000000	BCW_6461	*C. jejuni*	28	1,724,268	1,755
MJXK00000000	BCW_6462	*C. jejuni*	45	1,762,804	1,774
MJXL00000000	BCW_6463	*C. jejuni*	80	1,810,501	1,847
MJXM00000000	BCW_6464	*C. jejuni*	89	1,874,875	1,906
MJXN00000000	BCW_6465	*C. jejuni*	113	1,898,179	1,918
MJXO00000000	BCW_6466	*C. jejuni*	75	1,856,101	1,908
MJXP00000000	BCW_6467	*C. jejuni*	52	1,706,044	1,706
MJXQ00000000	BCW_6468	*C. jejuni*	44	1,745,966	1,746
MJYM00000000	BCW_6475	*C. jejuni*	133	1,715,549	1,732
MJYN00000000	BCW_6476	*C. jejuni*	49	1,750,597	1,751
MJWQ00000000	BCW_6871	*C. jejuni*	83	1,838,932	1,893
MJWR00000000	BCW_6872	*C. jejuni*	38	1,727,268	1,728
MJWS00000000	BCW_6873	*C. jejuni*	114	1,864,402	1,881
MJWT00000000	BCW_6874	*C. jejuni*	116	1,864,348	1,871
MJWU00000000	BCW_6875	*C. jejuni*	40	1,737,054	1,756
MJWV00000000	BCW_6876	*C. jejuni*	39	1,798,356	1,914
MJWW00000000	BCW_6877	*C. jejuni*	65	1,866,607	1,905
MJWX00000000	BCW_6878	*C. jejuni*	114	1,862,090	1,875
MJWY00000000	BCW_6879	*C. jejuni*	31	1,633,912	1,684
MJYO00000000	BCW_6880	*C. jejuni*	40	1,684,005	1,685
MJYQ00000000	BCW_6881	*C. jejuni*	90	1,789,448	1,773
MJYR00000000	BCW_6882	*C. jejuni*	85	1,886,467	1,891
MJYS00000000	BCW_6883	*C. jejuni*	82	1,870,773	1,908
MKEW00000000	BCW_6884	*C. jejuni*	111	1,874,853	1,925
MJYT00000000	BCW_6885	*C. jejuni*	85	1,745,151	1,777
MJYU00000000	BCW_6886	*C. jejuni*	114	1,737,339	1,758
MKEV00000000	BCW_6887	*C. jejuni*	141	1,802,256	1,801
MJYV00000000	BCW_6888	*C. jejuni*	81	1,676,843	1,691
MJWZ00000000	BCW_6889	*C. jejuni*	81	1,944,460	2,003
MJYW00000000	BCW_6891	*C. jejuni*	83	1,751,933	1,778
MJYX00000000	BCW_6893	*C. jejuni*	57	1,770,259	1,778
MJYY00000000	BCW_6896	*C. jejuni*	120	1,625,057	1,606
MJYZ00000000	BCW_6897	*C. jejuni*	27	1,713,241	1,717
MJZA00000000	BCW_6898	*C. jejuni*	36	1,668,807	1,665
MJZB00000000	BCW_6899	*C. jejuni*	35	1,674,680	1,665
MJZC00000000	BCW_6900	*C. jejuni*	60	1,839,405	1,838
MJZD00000000	BCW_6901	*C. jejuni*	172	1,671,632	1,678
MKEU00000000	BCW_6902	*C. jejuni*	37	1,706,600	1,714
MJZE00000000	BCW_6904	*C. jejuni*	71	1,719,544	1,739
MKAP00000000	BCW_6907	*C. jejuni*	55	1,829,263	1,849
MJZF00000000	BCW_6910	*C. jejuni*	47	1,817,981	1,839
MJZN00000000	BCW_6953	*C. jejuni*	40	1,700,213	1,723
MJZO00000000	BCW_6954	*C. jejuni*	81	1,881,118	1,881
MJZQ00000000	BCW_6956	*C. jejuni*	57	1,783,446	1,795
MJZT00000000	BCW_6959	*C. jejuni*	48	1,699,337	1,726
MJZZ00000000	BCW_7438	*C. jejuni*	73	1,950,893	2,009
MJVX00000000	BCW_3783	*C. lari*	23	1,493,439	1,495
MJVW00000000	BCW_3793	*C. lari*	25	1,492,968	1,492
MJWK00000000	BCW_4217	*C. lari*	21	1,491,293	1,492

a The average number of contigs, genome size, and number of coding sequences were 41.4, 1,828,002.30 bp, and 1,884 for *C. coli*; 63.8, 1,764,345.40 bp, and 1,792 for *C. jejuni*; and 23, 1,492,566.50 bp, and 1,493 for *C. lari*, respectively.

b CDSs, coding sequences.

### Accession number(s).

Sequences can be found in the 100K Project BioProject at the NCBI SRA BioProject and in the NCBI Genbank. Accession numbers are presented in Table 1.
